# Supplementary material for: 5-FU promotes stemness of colorectal cancer via p53-mediated WNT/β-catenin pathway activation
Source: Nat Commun. 2020 Oct 21;11:5321. doi: 10.1038/s41467-020-19173-2 (PMC7578039; doi:10.1038/s41467-020-19173-2)
Supplement: Supplementary file 1 — Supplementary Information [file 41467_2020_19173_MOESM1_ESM.pdf]

Supplementary Information

5-FU promotes stemness of colorectal cancer via p53-mediated WNT/β-catenin pathway activation

Cho and Ro et al.

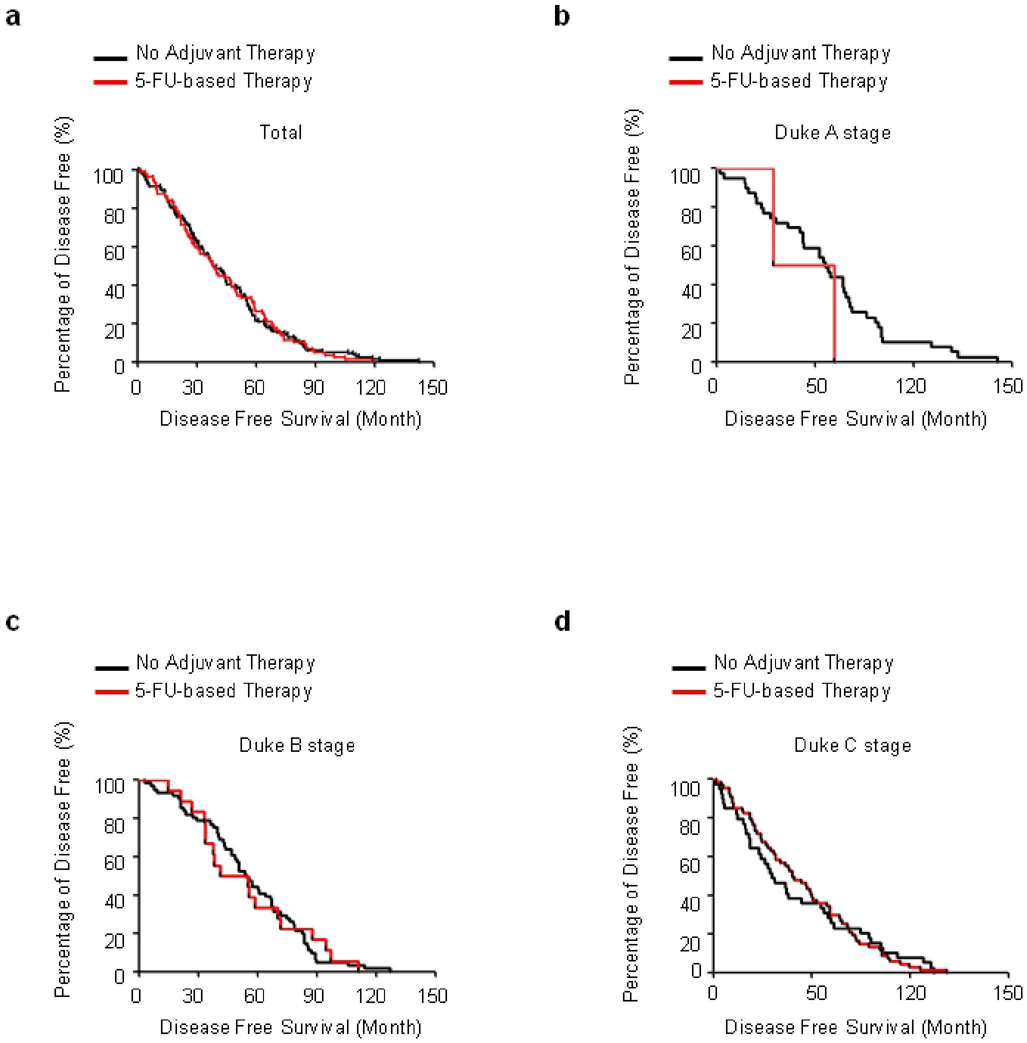
a
No Adjuvant Therapy
5-FU-based Therapy
Total
Percentage of Disease Free (%)
100
80
60
40
20
0
0
30
60
90
120
150
Disease Free Survival (Month)
b
No Adjuvant Therapy
5-FU-based Therapy
Duke A stage
Percentage of Disease Free (%)
100
80
60
40
20
0
0
50
120
150
Disease Free Survival (Month)
c
No Adjuvant Therapy
5-FU-based Therapy
Duke B stage
Percentage of Disease Free (%)
100
80
60
40
20
0
0
30
60
90
120
150
Disease Free Survival (Month)
d
No Adjuvant Therapy
5-FU-based Therapy
Duke C stage
Percentage of Disease Free (%)
100
80
60
40
20
0
0
50
120
150
Disease Free Survival (Month)

Supplementary Fig. 1

5-FU-based therapy does not improve disease-free survival of CRC patients.

a-d Disease-free survival rates in patients with differential stages after 5-FU-based treatment were analyzed based on the core data set (GSE14333). All stages (**a**), Duke A stage (**b**), Duke B stage (**c**), and Duke C stage (**d**) treated with or without 5-FU-based adjuvant chemotherapy were analyzed (log-rank test, Cox proportional regression).

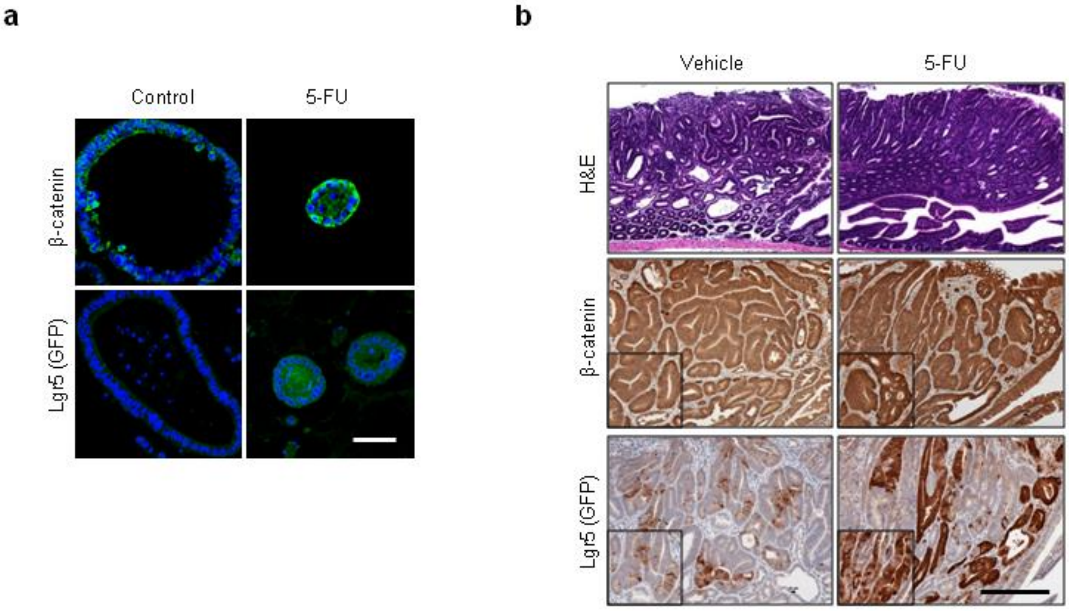
a
Control
5-FU
β-catenin
Lgr5 (GFP)
b
Vehicle
5-FU
H&E
β-catenin
Lgr5 (GFP)

Supplementary Fig. 2

5-FU activates WNT/β-catenin pathway and CSCs in murine CRC models.

a Confocal images of β-catenin (green) and GFP-Lgr5 (green) in *Apc*$^{Min/+}$*/K-Ras*^{G12D}*LA2/Lgr5*^{EGFP} intestinal tumor organoids with or without 5-FU treatment (1.5 μg/ml, 48 h). Scale bar=10 μm. **b** Hematoxylin and eosin staining and immunofluorescence staining of indicated proteins in intestinal sections of *Apc*$^{Min/+}$*/K-Ras*^{G12D}*LA2/Lgr5*^{EGFP} mice after treatment with vehicle or 5-FU (25 mg/kg, 3 times a week for 3 weeks). Scale bar=100 μm.

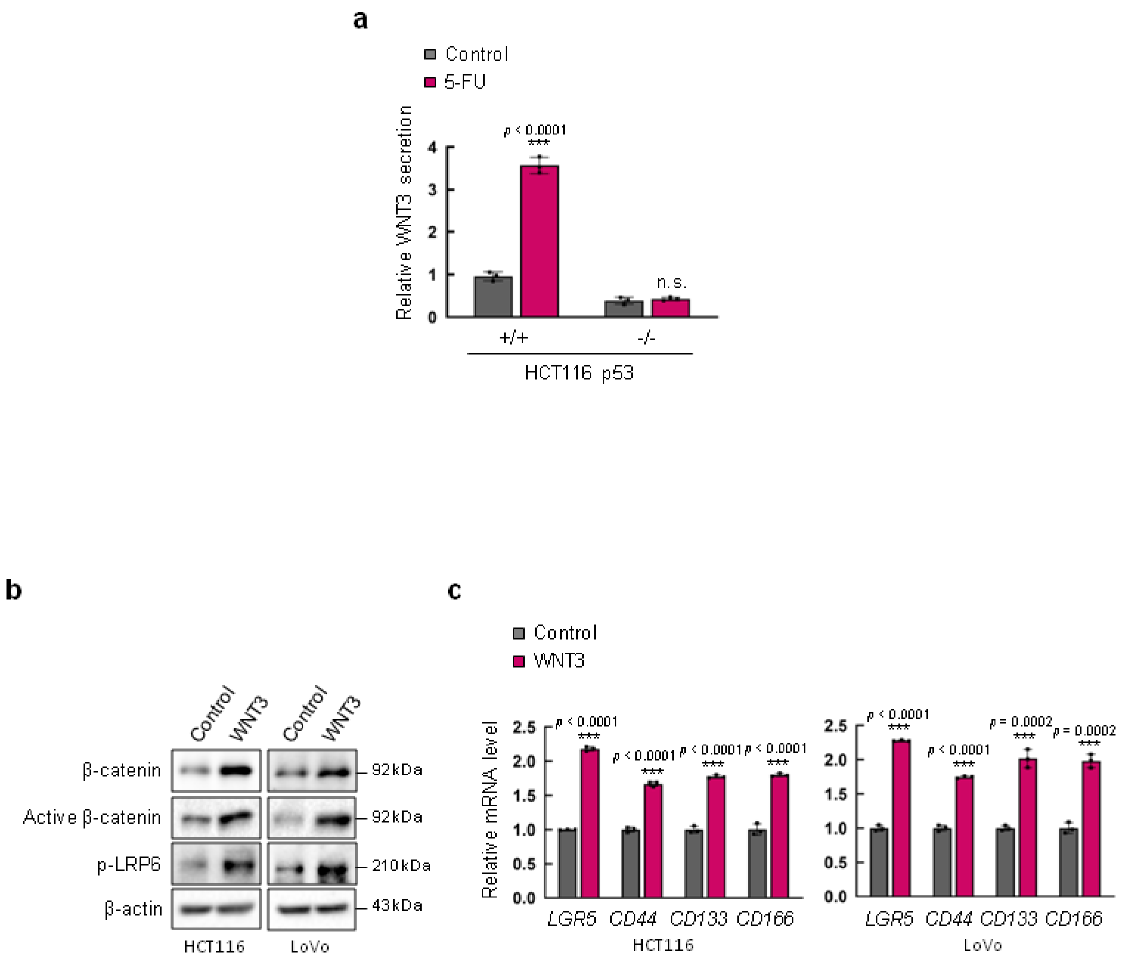
a
Control
5-FU
Relative WNT3 secretion
p < 0.0001
***
n.s.
+/+
-/-
HCT116 p53
b
Control
WNT3
Control
WNT3
β-catenin
Active β-catenin
p-LRP6
β-actin
92kDa
92kDa
210kDa
43kDa
HCT116
LoVo
c
Control
WNT3
Relative mRNA level
p < 0.0001
p = 0.0002
p = 0.0002
LGR5
CD44
CD133
CD166
HCT116
LoVo

Supplementary Fig. 3

5-FU induces p53-dependent WNT3 secretion in CRC cells with wild-type *p53.*

a ELISA analysis of WNT3 secretion in the culture medium of *p53* wild-type and *p53* knockout isogenic HCT116 cell lines grown with or without 5-FU treatment for 12 h (1.5 μg/ml). (n=3 biologically independent samples per group) **b** Immunoblot analyses of indicated proteins in HCT116 and LoVo cells with or without WNT3 treatment (50 ng/ml, 48 h). **c** Relative mRNA levels of *LGR5, CD44, CD133,* and *CD166* in HCT116 and LoVo CRC cells after WNT3 treatment (50 ng/ml, 48 h). (n=3 biologically independent samples per group) Data are mean ± s.d., two-sided Student's t-test, n.s. not significant, ** $p < 0.01$, ***$p < 0.001$. Source data are provided as a Source Data file.

a

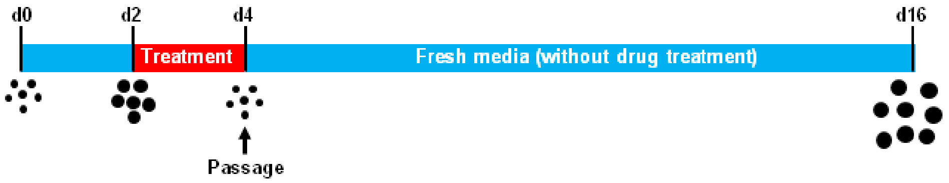
d0
d2
d4
d16
Treatment
Fresh media (without drug treatment)
Passage

b

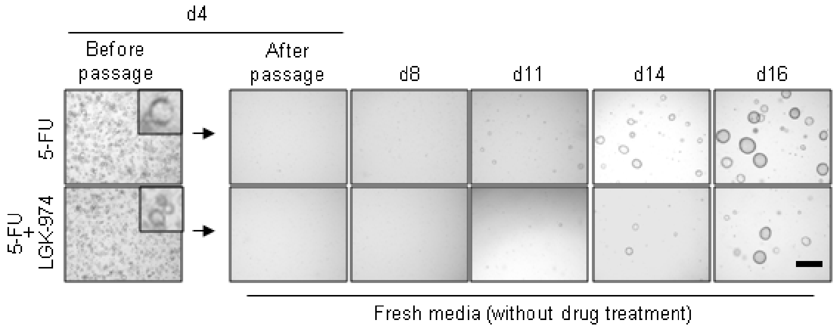
d4
Before passage
After passage
d8
d11
d14
d16
5-FU
5-FU + LGK-974
Fresh media (without drug treatment)

c

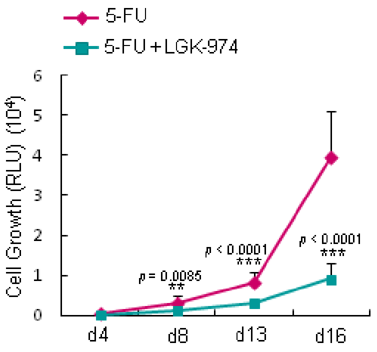
5-FU
5-FU+LGK-974
Cell Growth (RLU) (10⁴)
0
1
2
3
4
5
6
d4
d8
d13
d16
p = 0.0086
**
p < 0.0001
***
p < 0.0001
***

Supplementary Fig. 4

LGK-974 suppressed the clonogenicity of 5-FU-treated murine tumor organoids.

a-c Tumor organoids derived from $Apc^{Min/+}/Lgr5^{EGFP}$ mice were treated with 5-FU (1.5 µg/ml, 48 h) alone or co-treated with LGK-974 (5 µM, 48 h). After 48 h, tumor organoids were dissociated and passaged as single cells in fresh media without drug treatment, and the formation and growth of organoids were observed for 12 days. **a** scheme of the assay. **b** Representative bright-field images at each time point by EVOS microscope. Scale bar=650 µm. **c** Growth of tumor organoids derived from 5-FU alone or co-treated with LGK-974 $Apc^{Min/+}/Lgr5^{EGFP}$ tumor organoids were measured using Cell Titer-Glo® Luminescent Cell Viability Assay at indicated days. (n=9 in D4, n=8 in D8 and D13, and n=12 in D16 biologically independent samples per group) Data are mean ± s.d., two-sided Student's t-test, ** $p < 0.01$, ***$p < 0.001$. Source data are provided as a Source Data file.

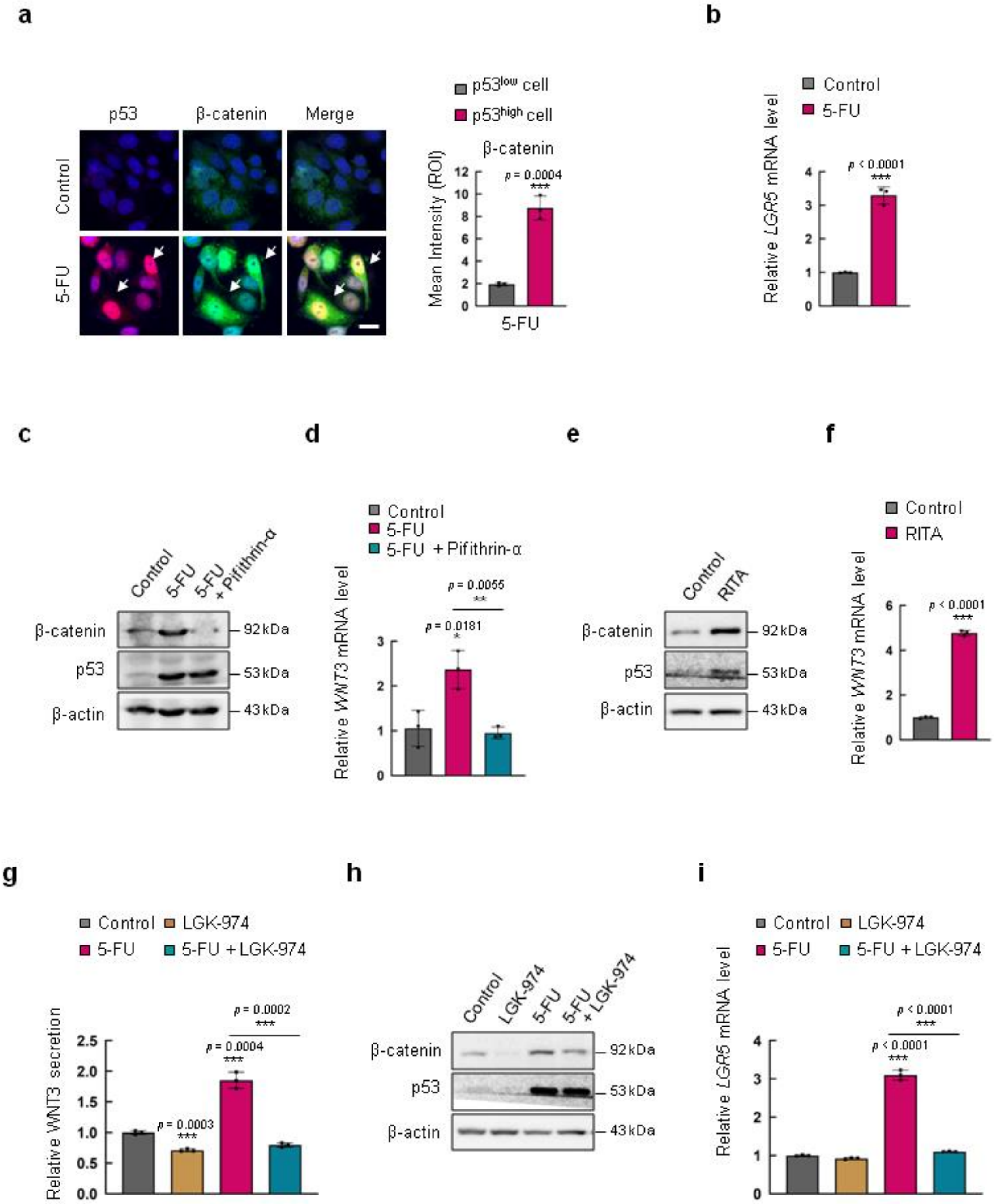

a
b
c
d
e
f
g
h
i
p53
β-catenin
Merge
Control
5-FU
p53low cell
p53high cell
β-catenin
Mean Intensity (ROI)
p = 0.0004
***
5-FU
Control
5-FU
Relative LGR5 mRNA level
p < 0.0001
***
Control
5-FU
5-FU + Pifithrin-α
β-catenin
p53
β-actin
92 kDa
53 kDa
43 kDa
Control
5-FU
5-FU + Pifithrin-α
Relative WNT3 mRNA level
p = 0.0055
**
p = 0.0181
*
Control
RITA
β-catenin
p53
β-actin
92 kDa
53 kDa
43 kDa
Control
RITA
Relative WNT3 mRNA level
p < 0.0001
***
Control
LGK-974
5-FU
5-FU + LGK-974
Relative WNT3 secretion
p = 0.0002
***
p = 0.0004
***
p = 0.0003
***
Control
LGK-974
5-FU
5-FU + LGK-974
β-catenin
p53
β-actin
92 kDa
53 kDa
43 kDa
Control
LGK-974
5-FU
5-FU + LGK-974
Relative LGR5 mRNA level
p < 0.0001
***
p < 0.0001
***

Supplementary Fig. 5

5-FU activates CSCs via p53-mediated *WNT3* transcription in LoVo CRC cells.

a Confocal images of immunofluorescence staining for indicated proteins in LoVo cells treated with or without 5-FU (1.5 μg/ml, 48 h) (left panel) and the mean intensity of β-catenin in $p53^{low}$ and $p53^{high}$ cells (right panel) (n=3 biologically independent samples per group). Arrows indicate cells highly induced p53 and β-catenin. Scale bar=20 μm. The mean intensity was measured by Zen software 3.1. **b** Relative mRNA expression levels of *LGR5* in LoVo cells treated or non-treated with 5-FU (1.5 μg/ml, 48 h). (n=3 biologically independent samples per group) **c** Immunoblot analyses of indicated proteins in LoVo cells treated without 5-FU, with 5-FU (1.5 μg/ml, 48 h), and with 5-FU (1.5 μg/ml, 48 h) and pifithrin-α (10 μM, 48 h). **d** Relative mRNA levels of *WNT3* in LoVo cells treated with or without treatment of 5-FU (1.5 μg/ml, 48 h) and pifithrin-α (10 μM, 48 h). (n=3 biologically independent samples per group) **e** Immunoblot analyses of indicated proteins in LoVo cells treated with or without treatment with RITA (5 μM, 48 h). **f** Relative mRNA expression levels of *WNT3* were measured by RT-PCR analyses with total RNA form LoVo cells treated with or without RITA (10 μM, 48 h). (n=3 biologically independent samples per group) **g** ELISA analyses was performed to detect WNT3 by using 12 h cultured medium obtained by growing LoVo cells with 5-FU (1.5 μg/ml) alone or co-treatment with 5-FU (1.5 μg/ml) and LGK-974 (5 μM). (n=3 biologically independent samples per group) **h** Immunoblots of indicated proteins in LoVo cells treated with 5-FU (1.5 μg/ml, 48 h) alone or co-treated with 5-FU (1.5 μg/ml, 48 h) and LGK-974 (5 μM, 48 h). **i** Relative mRNA levels of *LGR5* in LoVo cells treated with 5-FU (1.5 μg/ml, 48 h) alone or co-treated with 5-FU (1.5 μg/ml, 48 h) and LGK-974 (5 μM, 48 h). (n=3 biologically independent samples per group) Data are mean ± s.d., two-sided Student's t-test, * $p < 0.05$, ***$p < 0.001$. Source data are provided as a Source Data file.

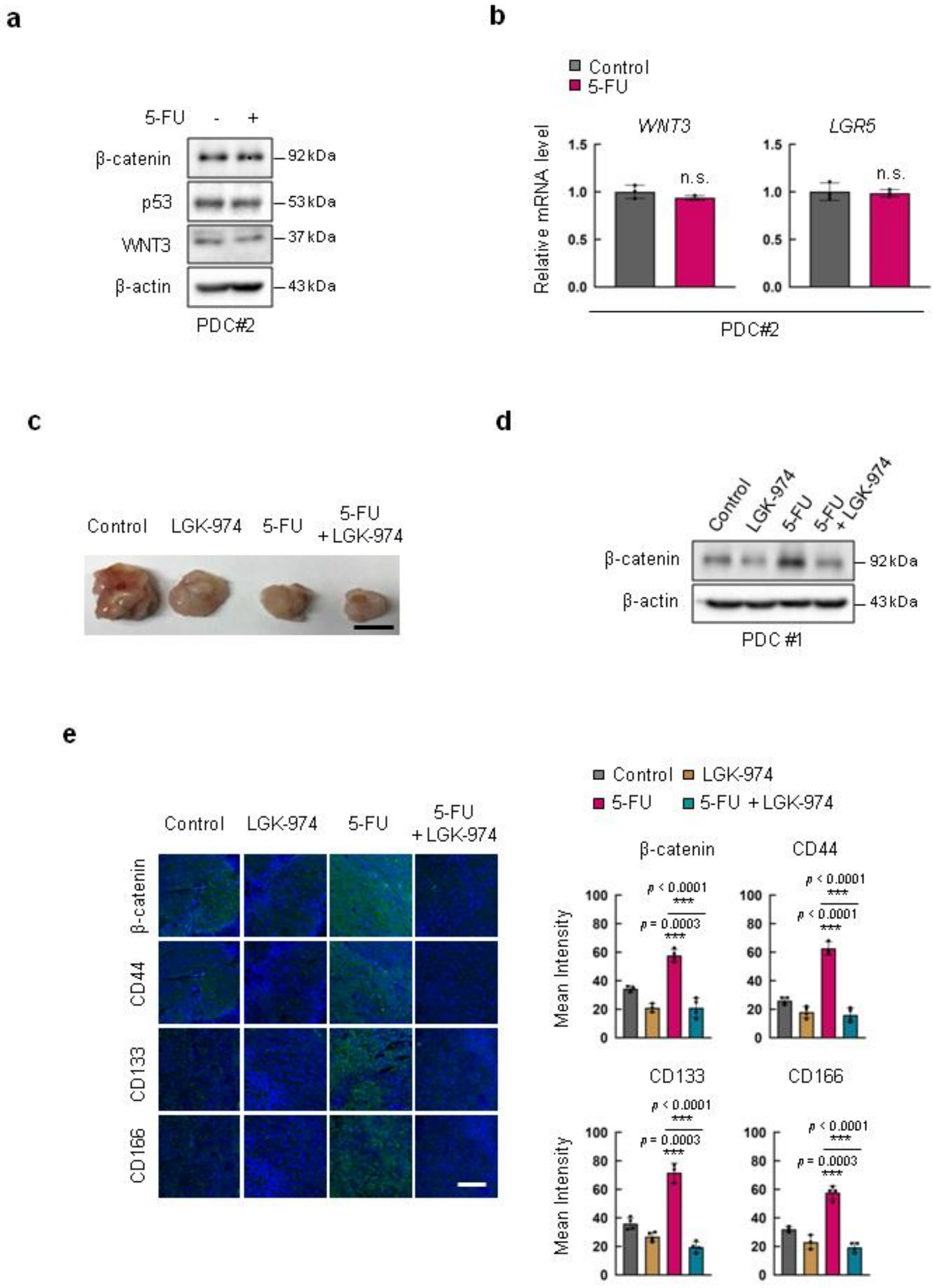
a
5-FU - +
β-catenin
p53
WNT3
β-actin
92 kDa
53 kDa
37 kDa
43 kDa
PDC#2
b
Control
5-FU
WNT3
LGR5
Relative mRNA level
1.5
1.0
0.5
0.0
n.s.
PDC#2
c
Control
LGK-974
5-FU
5-FU + LGK-974
d
Control
LGK-974
5-FU
5-FU + LGK-974
β-catenin
β-actin
92 kDa
43 kDa
PDC #1
e
Control
LGK-974
5-FU
5-FU + LGK-974
β-catenin
CD44
CD133
CD166
Mean Intensity
p < 0.0001
p = 0.0003
***
100
80
60
40
20
0

Supplementary Fig. 6

WNT inhibitor suppresses 5-FU-induced activation of the WNT/β-catenin pathway in PDCs.

a Immunoblots of indicated proteins in PDC#2 harboring mutant *p53* after treatment of 5-FU (1.5 μg/ml, 48 h). **b**. Relative mRNA levels of *WNT3* and *LGR5* in PDC#2 with or without 5-FU treatment (1.5 μg/ml, 48 h). (n=3 biologically independent samples per group) **c-e** *p53* wild-type PDC#1 xenograft mice were treated with 5-FU (25 mg/kg) or LGK-974 (5 mg/kg) alone or co-treated with 5-FU (25 mg/kg) and LGK-974 (5 mg/kg) for 16 days and tumors were extracted and analyzed. **c** Representative gross images of extracted tumors. Scale bars=10 mm. **d** Immunoblots of indicated proteins in PDC#1 xenograft tumors. **e** Immunocytochemical analysis of indicated proteins in sections of PDC#1 xenograft (left panel) and quantification of mean intensities (right panel). Scale bars=100 μm. The mean intensities were measured by Zen software 3.1. (β-catenin and CD166 n=3 in Control and LGK-974 group and n=4 in 5-FU and 5-FU+LGK-974 group, CD44 and CD133 n=4 in Control, LGK-974 and 5-FU+LGK-974 group, and n=3 in 5-FU group) Data are mean ± s.d., two-sided Student's t-test, n.s. not significant, ***$p < 0.001$. Source data are provided as a Source Data file.

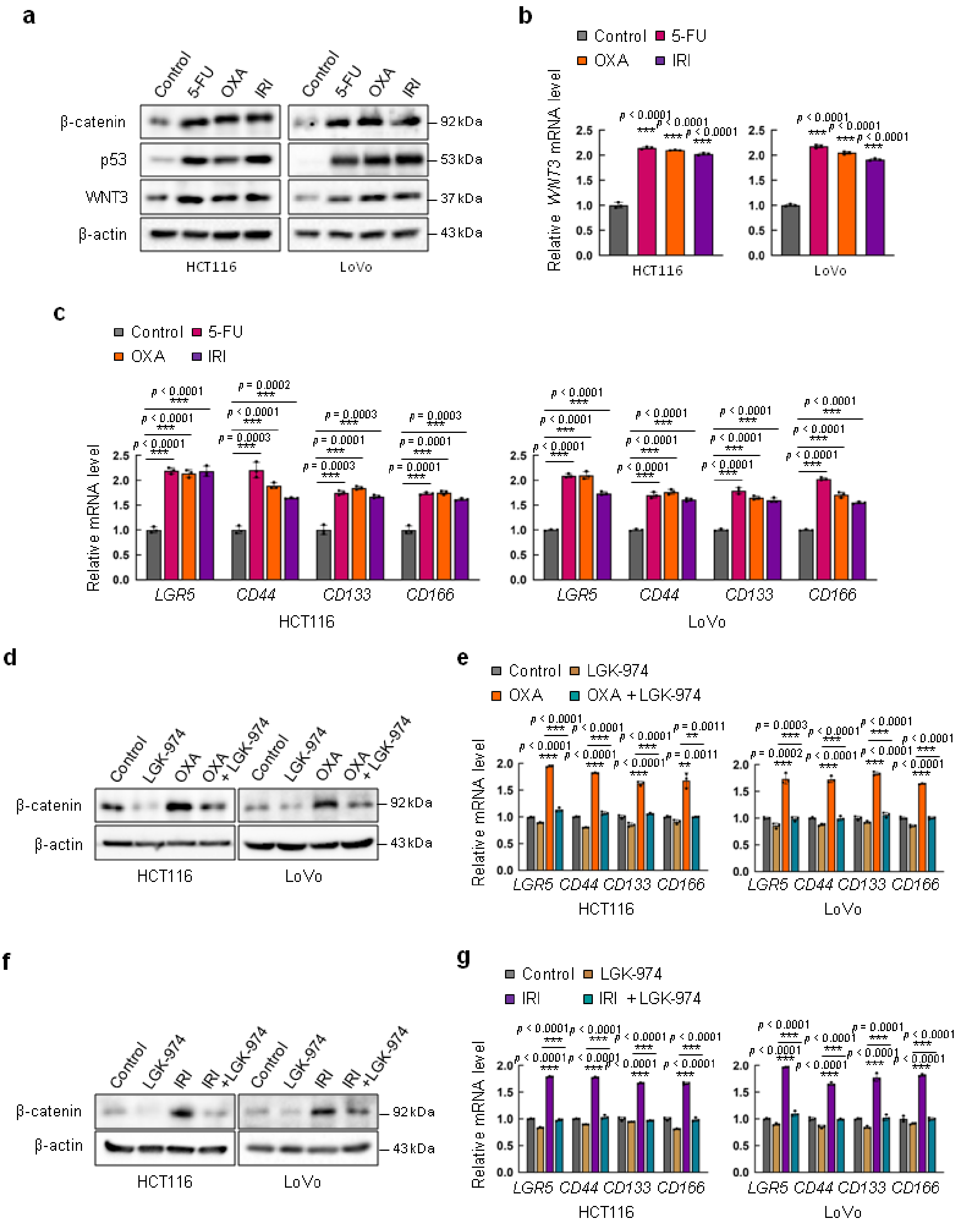
a
Control
5-FU
OXA
IRI
β-catenin
p53
WNT3
β-actin
92 kDa
53 kDa
37 kDa
43 kDa
HCT116
LoVo
b
Control
5-FU
OXA
IRI
Relative WNT3 mRNA level
HCT116
LoVo
c
Control
5-FU
OXA
IRI
Relative mRNA level
LGR5
CD44
CD133
CD166
HCT116
LoVo
d
Control
LGK-974
OXA
OXA + LGK-974
β-catenin
β-actin
92 kDa
43 kDa
HCT116
LoVo
e
Control
LGK-974
OXA
OXA + LGK-974
Relative mRNA level
LGR5 CD44 CD133 CD166
HCT116
LoVo
f
Control
LGK-974
IRI
IRI + LGK-974
β-catenin
β-actin
92 kDa
43 kDa
HCT116
LoVo
g
Control
LGK-974
IRI
IRI + LGK-974
Relative mRNA level
LGR5 CD44 CD133 CD166
HCT116
LoVo

Supplementary Fig. 7

Combined inhibition of WNT suppresses the chemotherapy-induced CSC activation.

a-c HCT116 and LoVo cells were treated with 5-FU (1.5 μg/ml, 48 h), oxaliplatin (2.5 μg/ml, 48 h), or irinotecan (10 μM, 48 h). **a** Immunoblots of indicated proteins in HCT116 and LoVo cells after indicated treatment. **b, c** Relative mRNA levels of *WNT3* (**b**) and *LGR5*, *CD44, CD133*, and *CD166* CSC markers (**c**) in HCT116 and LoVo after indicated treatment. (n=3 biologically independent samples per group) **d, f** Immunoblots of indicated proteins in HCT116 and LoVo cells treated with oxaliplatin (2.5 μg/ml, 48 h) alone or co-treated with oxaliplatin (2.5 μg/ml, 48 h) and LGK-974 (5 μM, 48 h) (**d**), and irinotecan (10 μM, 48 h) alone or co-treated with irinotecan and LGK-974 (5 μM, 48 h) (**f**). **e, g** Relative mRNA levels of *LGR5*, *CD44*, *CD133*, and *CD166* in HCT116 and LoVo cells after treatment with oxaliplatin (2.5 μg/ml, 48 h) alone or co-treatment with oxaliplatin (2.5 μg/ml, 48 h) and LGK-974 (5 μM, 48 h) (**e**), and irinotecan (10 μM, 48 h) alone or co-treated with irinotecan and LGK-974 (5 μM, 48 h) (**g**). (n=3 biologically independent samples per group) Data are mean ± s.d., two-sided Student's t-test, *p < 0.05, **p < 0.01, ***p < 0.001. Source data are provided as a Source Data file.

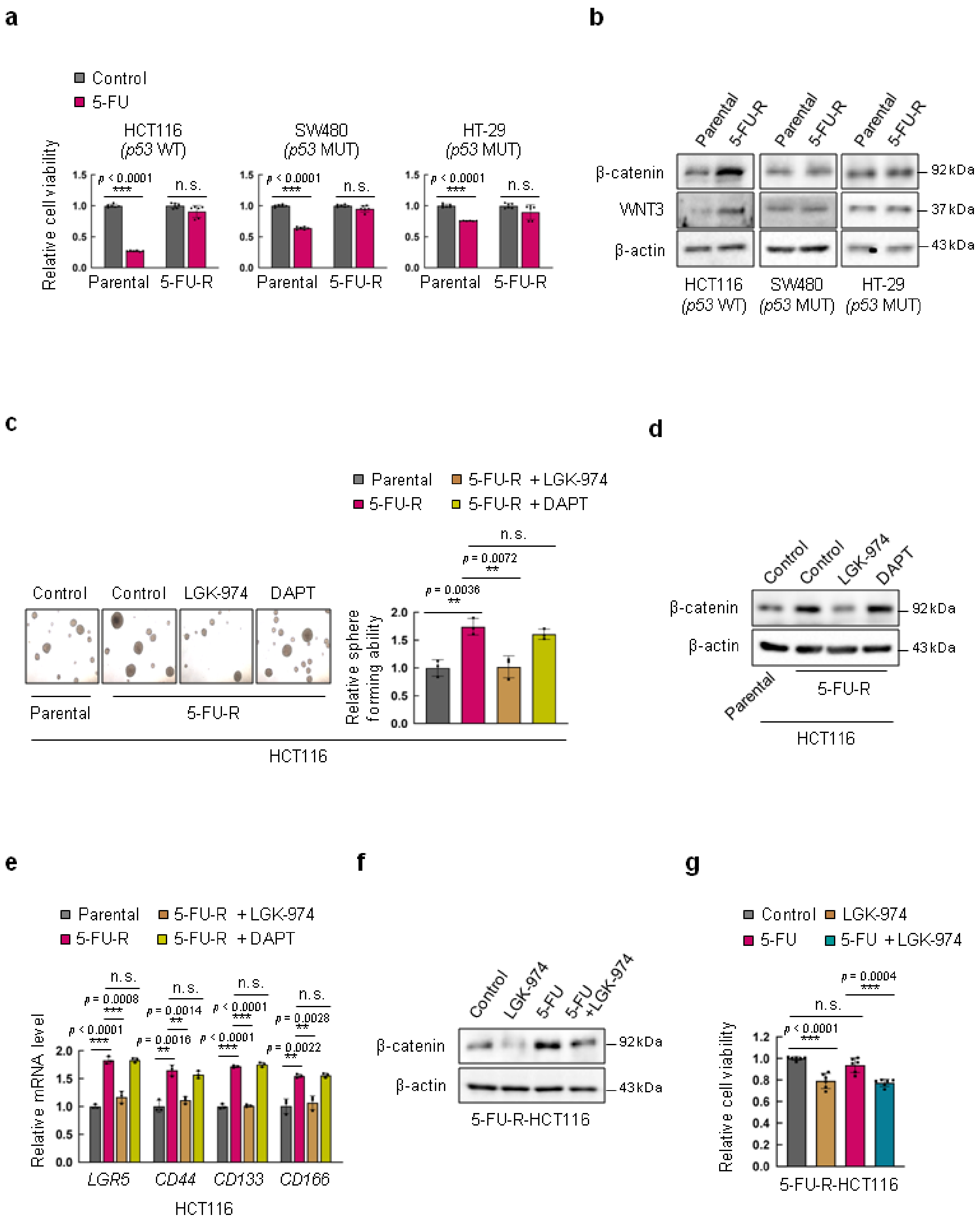

a
Control
5-FU
HCT116 (p53 WT)
SW480 (p53 MUT)
HT-29 (p53 MUT)
Relative cell viability
p < 0.0001
***
n.s.
Parental
5-FU-R
b
Parental
5-FU-R
β-catenin
WNT3
β-actin
92 kDa
37 kDa
43 kDa
HCT116 (p53 WT)
SW480 (p53 MUT)
HT-29 (p53 MUT)
c
Parental
5-FU-R
5-FU-R +LGK-974
5-FU-R +DAPT
Control
LGK-974
DAPT
Relative sphere forming ability
p = 0.0036
p = 0.0072
HCT116
d
β-catenin
β-actin
92 kDa
43 kDa
e
Relative mRNA level
LGR5
CD44
CD133
CD166
p = 0.0008
p = 0.0014
p = 0.0016
p = 0.0028
p = 0.0022
f
5-FU
5-FU +LGK-974
5-FU-R-HCT116
g
p = 0.0004

Supplementary Fig. 8

WNT inhibitor suppresses the cancer stemness of 5-FU-R-HCT116 cells.

a MTT analyses using 5-FU-resistant HCT116, SW480, and HT-29 cells and their parental counterparts after treatment of 5-FU (1.5 μg/ml, 72 h). (n=6 biologically independent samples per group) **b** Immunoblots of indicated proteins in 5-FU-resistant HCT116, SW480, and HT29 cells and their parental counterparts. **c-e** Spheroids derived from parental- and 5-FU-R-HCT116 cells were cultured for 5 days with or without treatment of LGK-974 (5 μM) and DAPT (5 μM). **c** Bright field images of spheroids derived from parental- and 5-FU-R-HCT116 cells with indicated treatment (left panel). Right panel represents quantification results of their spheroid forming abilities as analyzed by using image J software. (n=3 biologically independent samples per group) **d** Immunoblots of indicated proteins in spheroids derived from parental- and 5-FU-R-HCT116 cells with indicated treatment. **e** Relative mRNA levels of *LGR5, CD44, CD133,* and *CD166* in parental- and 5-FU-R-HCT116 spheroids with indicated treatment. (n=3 biologically independent samples per group) **f** Immunoblots of indicated proteins in 5-FU-R-HCT116 cells treated with or without 5-FU (1.5 μg/ml, 48 h) alone, or co-treated with 5-FU (1.5 μg/ml, 48 h) and LGK-974 (5 μM, 48 h). **g** MTT analyses of 5-FU-R-HCT116 cells treated with or without 5-FU (1.5 μg/ml, 72 h) alone, or co-treated with 5-FU (1.5 μg/ml, 72 h) and LGK-974 (5 μM, 72 h). (n=6 biologically independent samples per group) Data are mean ± s.d., two-sided Student's t-test, n.s. not significant, *$p < 0.05$, **$p < 0.01$, ***$p < 0.001$. Source data are provided as a Source Data file.

Supplementary Table 1. Genetic profiles of CRC patients.

**Case**	***APC***	***KRAS***	***TP53***	***EGFR***	***PIK3CA***
PDC#1	WT	G12D	WT	WT	E545K
PDC#2	R1450*	WT	R248Q	WT	H1047R
PDC#3	WT	G12D	WT	WT	WT
PDTO#1	R216*	G12S	G245S	R521K	WT
PDTO#2	G1303*	WT	WT	R521K	WT
PDTO#3	R302*	G12D	WT	R521K	WT

*=truncated mutation
