# Supplementary material for: 5-FU promotes stemness of colorectal cancer via p53-mediated WNT/β-catenin pathway activation
Source: Nat Commun. 2020 Oct 21;11:5321. doi: 10.1038/s41467-020-19173-2 (PMC7578039; doi:10.1038/s41467-020-19173-2)
Supplement: Supplementary file 3 — Description of Additional Supplementary Files [file 41467_2020_19173_MOESM3_ESM.pdf]

Description of Additional Supplementary Files

File Name: Supplementary Movie 1
Description: Lgr5 (GFP) expression in murine CRC tumor organoid. Video showing the expression of Lgr5 (GFP)+ CSCs in un-treated control group for 48 h.

File Name: Supplementary Movie 2
Description: Lgr5 (GFP) enrichment by 5-FU in murine CRC tumor organoid. Video showing the activation and enrichment of Lgr5 (GFP)+ CSCs during 48 h treatment of 5-FU.
