# Supplementary material for: 5-FU promotes stemness of colorectal cancer via p53-mediated WNT/β-catenin pathway activation
Source: Nat Commun. 2020 Oct 21;11:5321. doi: 10.1038/s41467-020-19173-2 (PMC7578039; doi:10.1038/s41467-020-19173-2)
Supplement: Supplementary file 6 — Reporting Summary [file 41467_2020_19173_MOESM6_ESM.pdf]

nature research

Corresponding author(s): Kang-Yell Choi

Last updated by author(s): Aug 30, 2020

Reporting Summary

Nature Research wishes to improve the reproducibility of the work that we publish. This form provides structure for consistency and transparency in reporting. For further information on Nature Research policies, see Authors & Referees and the Editorial Policy Checklist .

Statistics

For all statistical analyses, confirm that the following items are present in the figure legend, table legend, main text, or Methods section.

n/a	Confirmed	
☐	☒	The exact sample size (*n*) for each experimental group/condition, given as a discrete number and unit of measurement
☐	☒	A statement on whether measurements were taken from distinct samples or whether the same sample was measured repeatedly
☐	☒	The statistical test(s) used AND whether they are one- or two-sided<br>*Only common tests should be described solely by name; describe more complex techniques in the Methods section.*
☒	☐	A description of all covariates tested
☒	☐	A description of any assumptions or corrections, such as tests of normality and adjustment for multiple comparisons
☐	☒	A full description of the statistical parameters including central tendency (e.g. means) or other basic estimates (e.g. regression coefficient) AND variation (e.g. standard deviation) or associated estimates of uncertainty (e.g. confidence intervals)
☐	☒	For null hypothesis testing, the test statistic (e.g. *F*, *t*, *r*) with confidence intervals, effect sizes, degrees of freedom and *P* value noted<br>*Give P values as exact values whenever suitable.*
☒	☐	For Bayesian analysis, information on the choice of priors and Markov chain Monte Carlo settings
☒	☐	For hierarchical and complex designs, identification of the appropriate level for tests and full reporting of outcomes
☒	☐	Estimates of effect sizes (e.g. Cohen's *d*, Pearson's *r*), indicating how they were calculated

Our web collection on statistics for biologists contains articles on many of the points above.

Software and code

Policy information about availability of computer code

Data collection	Western data were collected by LAS-4000 (GE health care).<br>IHC images were collected by LSM510 (Carl Zeiss).<br>Real-time PCR data were collected by Rotor-Gene Q (Qiagen).<br>Luciferase data were collected by Fluostar (BMG LABTECH).<br>Immunoblots were detected by LAS-4000 (Fuji Film) and ChemiDoc XRS+ System (Bio-Rad)
Data analysis	MultiGauge software V3.0 (Fuji) and Image Lab Software V6.0.1 (Bio-Rad) were used for immunoblot analyses.<br>Zen software (Carl Zeiss) was used for immunofluorescence confocal microscopy analyses (V3.1).<br>Prism software (GraphPad) was used for statistical analyses (V8.4.3).<br>Image J software was used for quantification analyses (V1.46)

For manuscripts utilizing custom algorithms or software that are central to the research but not yet described in published literature, software must be made available to editors/reviewers. We strongly encourage code deposition in a community repository (e.g. GitHub). See the Nature Research guidelines for submitting code & software for further information.

Data

Policy information about availability of data

All manuscripts must include a data availability statement. This statement should provide the following information, where applicable:

- Accession codes, unique identifiers, or web links for publicly available datasets
- A list of figures that have associated raw data
- A description of any restrictions on data availability

Disease-free survival rate data in patients with differential stages after 5-FU-based treatment discussed in this publication have been obtained from publicly available source in NCBI's Gene Expression Omnibus (Jorissen et al., 2009) and is accessible through GEO series accession number GSE14333. The source data underlying Figs. 1a-f, 2b, 2d, 2f, 3b, 3g, 3h, 4a-i, 5a, 5b, 5d, 5e, 5h, 5i, 6b, 6c, 6e-i and Supplementary Figs. 1, 3a, 3c, 4c, 5a, 5b, 5d, 5f, 5g, 5i, 6b, 6e, 7b, 7c, 7e, 7g,

8a, 8c, 8e, and 8g are provided as a Source Data file. All the other data supporting the findings of this study are available within the article and its supplementary information files and from the corresponding author upon reasonable request. A reporting summary for this article is available as a Supplementary Information file. Source data are provided with this paper.

Field-specific reporting

Please select the one below that is the best fit for your research. If you are not sure, read the appropriate sections before making your selection.

- [x] Life sciences
- [ ] Behavioural & social sciences
- [ ] Ecological, evolutionary & environmental sciences

For a reference copy of the document with all sections, see nature.com/documents/nr-reporting-summary-flat.pdf

Life sciences study design

All studies must disclose on these points even when the disclosure is negative.

Sample size	Sample size was chosen to assure reproducibility of the experiments in accordance with the replacement, reduction and refinement principles of animal ethics regulation. The statistical analyses were obtained using student t-test and the values represents means plus minus standard deviations or standard errors of the means.
Data exclusions	No samples or analyses were excluded from any experimental data presented in the manuscript
Replication	All experimental findings were reliably reproducible at least 3 biological independent experiments.
Randomization	All mice were age- and sex-matched (male mice) and then randomized into different experimental groups.
Blinding	The investigators were not blinded for group allocation during experiments, as the same investigator both planned and performed the experiment. Conclusions were made based on the quantitative parameters and statistical significance of the data, and thus on experimental observations, independent of blinding.

Reporting for specific materials, systems and methods

We require information from authors about some types of materials, experimental systems and methods used in many studies. Here, indicate whether each material, system or method listed is relevant to your study. If you are not sure if a list item applies to your research, read the appropriate section before selecting a response.

Materials & experimental systems

n/a	Involved in the study	
[ ]	[x]	Antibodies
[ ]	[x]	Eukaryotic cell lines
[x]	[ ]	Palaeontology
[ ]	[x]	Animals and other organisms
[ ]	[x]	Human research participants
[x]	[ ]	Clinical data

Methods

n/a	Involved in the study	
[x]	[ ]	ChIP-seq
[x]	[ ]	Flow cytometry
[x]	[ ]	MRI-based neuroimaging

Antibodies

Antibodies used

Immunoblotting
Mouse anti-p53 (Santa Cruz Biotechnology, DO-1, sc-126)
Rabbit anti-p21 (Santa Cruz Biotechnology, F-5, sc-6246)
Rabbit anti-β-catenin (Santa Cruz Biotechnology, H-102, sc-7199)
Rabbit anti-active β-catenin (Sigma-Aldrich, 8E7, #05-665)
Rabbit anti-Wnt3 (abcam, synthetic peptide within aa250-350 of mouse Wnt3, ab32249)
Rabbit anti-pLRP6 (Cell signaling Technology, ser1490, #2568)
Mouse anti-β-actin (Santa Cruz Biotechnology, C4, sc-47778)
Horseradish peroxidase-conjugated anti-mouse (Cell Signaling Technology, #7076) or anti-rabbit (Bio-Rad, #1706515) secondary antibodies.

Immunohistochemistry or Immunocytochemistry
Rabbit anti-β-catenin (Abcam, synthetic peptide within C-term of human β-catenin, ab2365)
Rabbit anti-CD44 (Proteintech, Ag8143, #15675-1-AP)
Mouse anti-CD133 (Miltenyi Biotec, AC133, #130-108-062)
Rabbit anti-CD166 (Abbiotec, Synthetic peptide within C-term region of human CD166, #251619).

	Mouse anti-GFP (Santa Cruz Biotechnology, F56-6A1, sc53882)<br>Rabbit anti-Wnt3 (abcam, synthetic peptide within aa250-350 of mouse Wnt3, ab32249)<br>Goat anti-mouse Alexa Fluor 488 (Life Technologies, A11008) or anti-rabbit Alex Fluor 555 (Life Technologies, A21428)<br><br>Antibodies used in ELISA<br>Wnt3 antibody-bound 96 wells, Biotin-conjugated antibody and Avidin-HRP antibody (MyBioSource, #MBS919345)
Validation	All antibodies use in this study are commercially available and validated in the literature as cited on the manufacture's websites.

Eukaryotic cell lines

Policy information about cell lines

Cell line source(s)	LoVo, HCT116, RKO, SW48, DLD-1, SW480, SW620, HT29, and WiDr were purchased from American Type Culture Collection (ATCC). P53 wild-type (p53+/+) and null (p53-/-) and p21 wild-type (p21+/+) and null (p21-/-) isogenic HCT 116 cell lines were provided by B. Vogelstein (John Hopkins oncology center, MD).
Authentication	HCT116, LoVo, RKO, SW480, DLD-1, SW48 CRC cell lines used in this study were authenticated.<br>SW620, WiDr, HT29, p53 wildtype and null and p21 wildtype and null isogenic HCT 116 cell lines were not authenticated.
Mycoplasma contamination	All cell lines teated negative for mycoplasma contamination
Commonly misidentified lines<br>(See ICLAC register)	None.

Animals and other organisms

Policy information about studies involving animals; ARRIVE guidelines recommended for reporting animal research

Laboratory animals	C57BL/6J-ApcMin/+ ; B6.129S-Krastm3Tyj ; B6N.129P2-Axin2tm1Wbm/J; Lgr5EGFP-IRES-creERT2, male, 12 weeks old mice. Athymic nu/nu mice (for cell line) and NOD SCID mice (for PDC), male, 5 weeks old mice. Mice were housed filter-topped shoebox cages with a MSRS environmental control system. Room temperature was maintained at 24 °C with a relative humidity of approximately 40% to 60%.
Wild animals	The study did not involve wild animals.
Field-collected samples	The study did not involve samples collected from the field.
Ethics oversight	All animal experiments were performed in accordance with the guidelines of the Korean Food and Drug Administration. Protocols were reviewed and approved by the Institutional Animal Care and Use Committee (IACUC) of Yonsei University.

Note that full information on the approval of the study protocol must also be provided in the manuscript.

Human research participants

Policy information about studies involving human research participants

Population characteristics	We included genetic profiles of patients related with this study in Supplementary table 1.
Recruitment	We recruited participants who had a colon biopsy in Yonsei medical school with patients' consent.
Ethics oversight	Archieved identified samples were used. Originally, informed consents was obtained from all patients under the oversight of Institutional Review Board (IRB) of University of Yonsei university. The entire experimental protocol was conducted in compliance with institutional guidelines.

Note that full information on the approval of the study protocol must also be provided in the manuscript.
